# Supplementary material for: A molecular switch from STAT2-IRF9 to ISGF3 underlies interferon-induced gene transcription
Source: Nat Commun. 2019 Jul 2;10:2921. doi: 10.1038/s41467-019-10970-y (PMC6606597; doi:10.1038/s41467-019-10970-y)
Supplement: Supplementary file 1 — Supplementary Information [file 41467_2019_10970_MOESM1_ESM.pdf]

A molecular switch from STAT2-IRF9 to ISGF3 underlies interferon-induced gene expression

Platanitis et al.

Supplementary Fig.1

1) Expression analysis

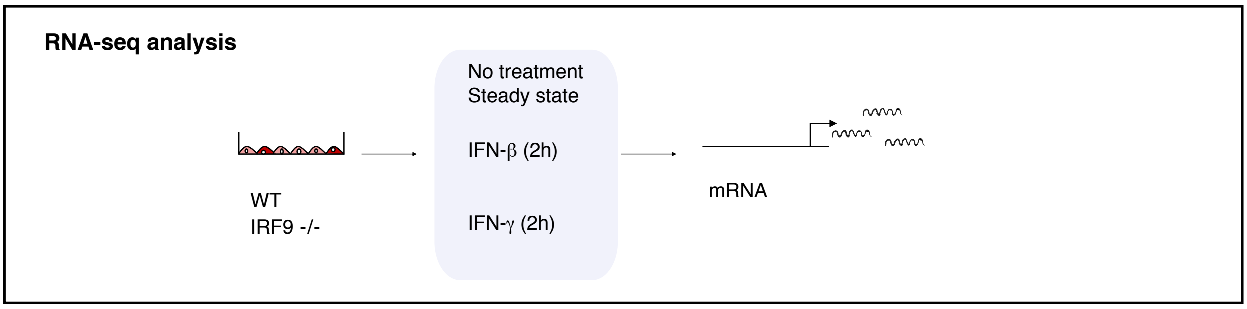

2) Transcription factor complexes

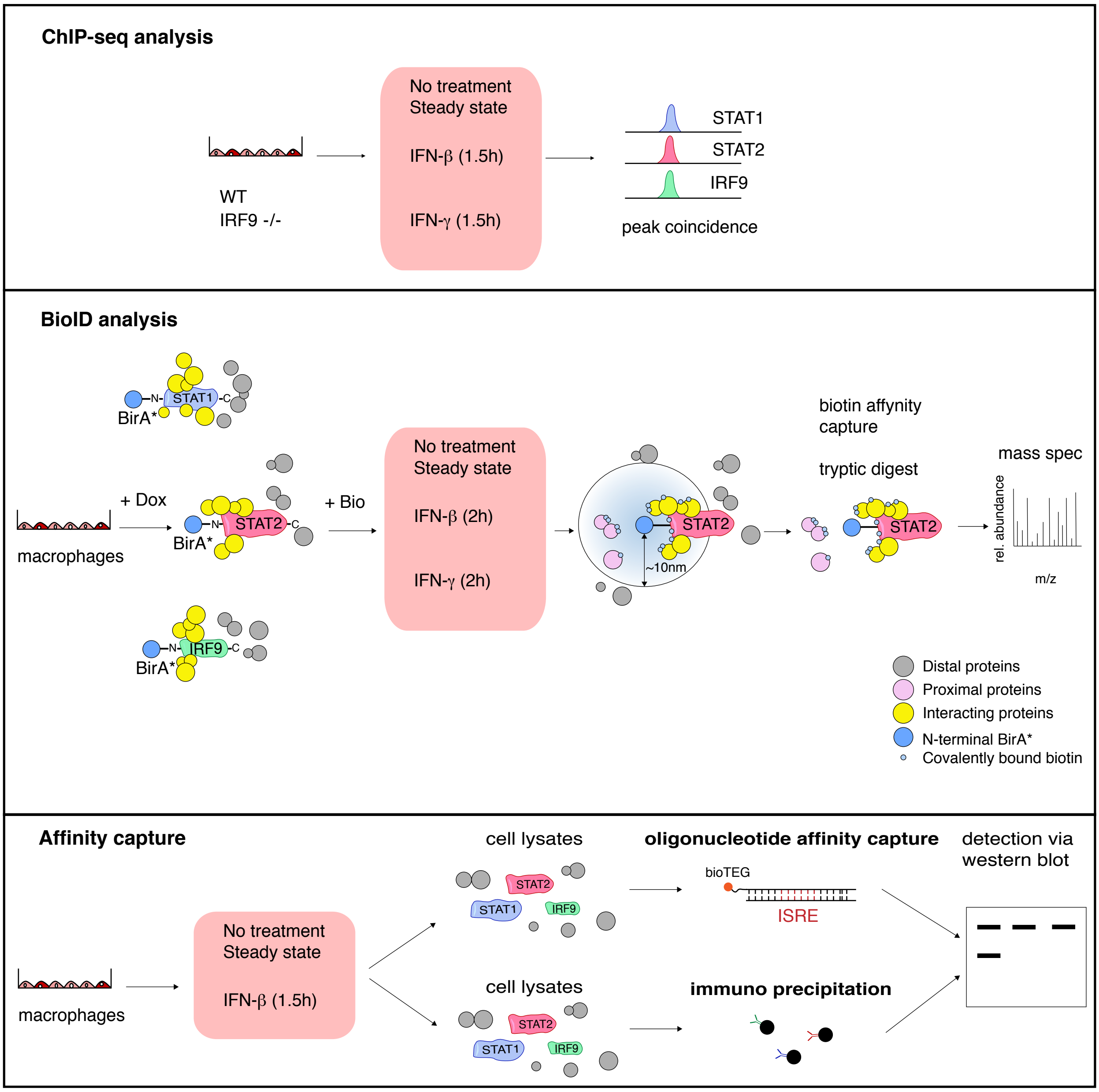

Supplementary Figure 1. Experimental workflow.

Supplementary Fig.2

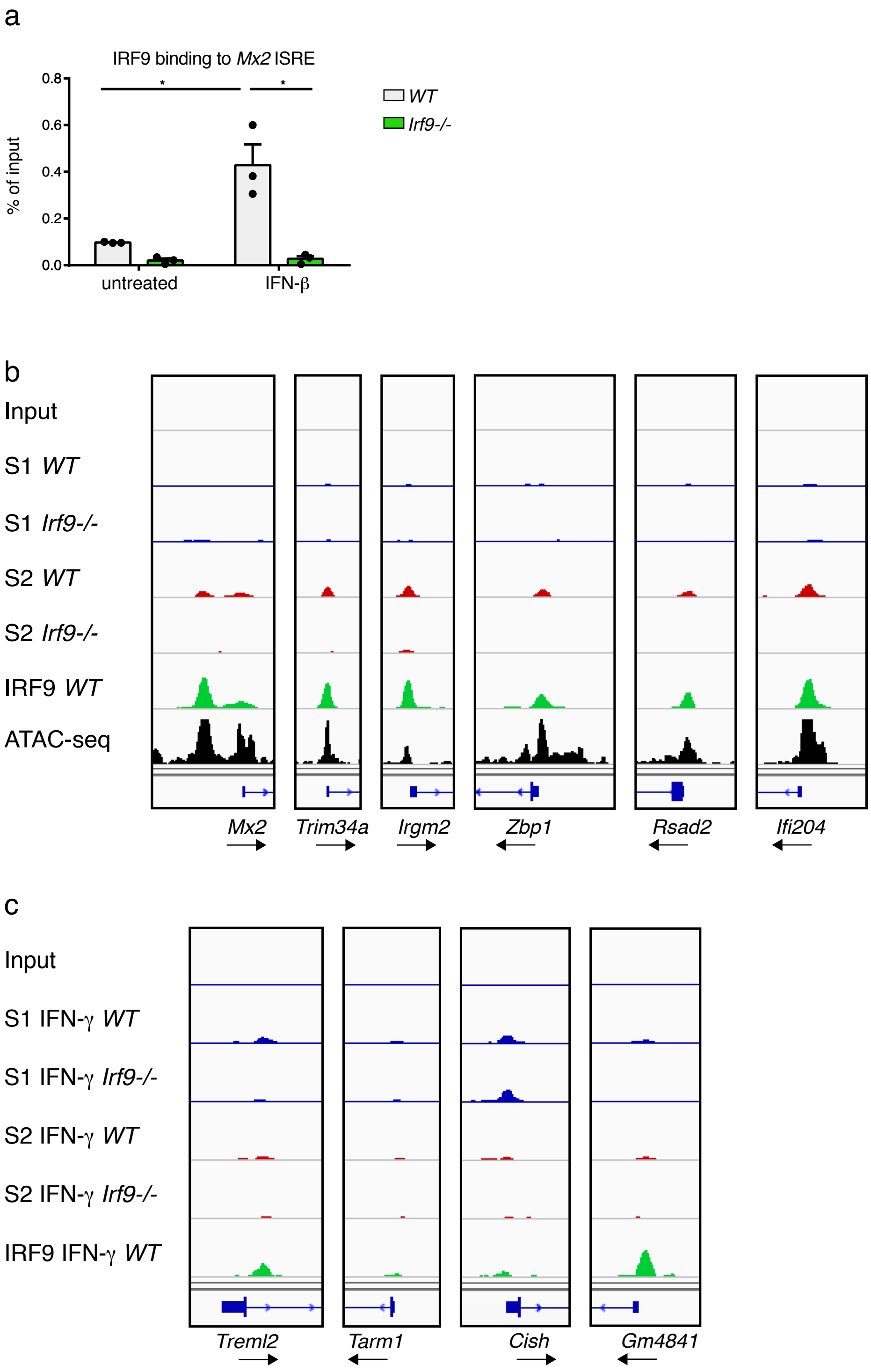

Supplementary Figure 2. IRF9 binding to promoters. a) Validation of the mIRF9 antibody by site-directed ChIP. IFN-β stimulated binding of IRF9 to the ISRE sequences of *Mx2* was analyzed using BMDM of *WT* and *Irf9-/-* (IRF9-/-) mice. Cells were treated with IFN-β for 1.5h. Data represent mean and SEM values of three independent experiments. P-values were calculated using the paired ratio t-test (*$P \leq 0.05$; **$P \leq 0.01$, ***$P \leq 0.001$). **b, c)** Representative genome browser tracks for transcription factor binding at ISG loci (scale 0-150) derived from ChIP-seq data described in the legend of Fig. 2. Data were obtained using *WT* or *Irf9-/-* BMDM treated with either IFN-β (1.5h) or IFN-γ (1.5h). Tracks show binding of STAT1 (S1/blue), STAT2 (S2/red) and IRF9 (green) or control input. Regulatory chromatin sites from ATAC-seq for untreated BMDM were obtained from[1] as described in the legend of Fig. 2.

Supplementary Fig.3

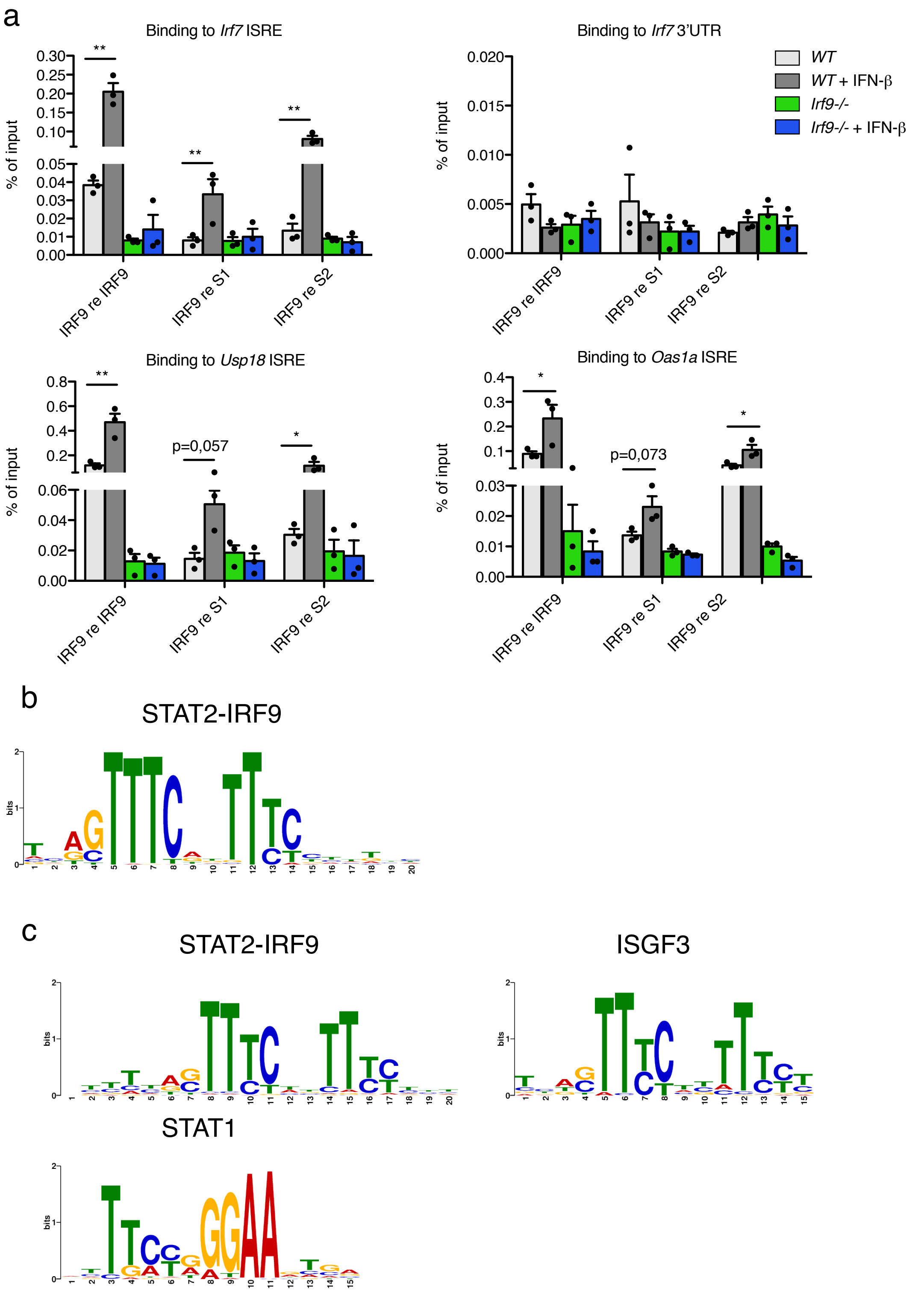

Supplementary Figure 3. a) Simultaneous association of STAT1, STAT2 and IRF9 at ISRE sites analyzed by ChIP-reChIP. BMDM of *wild-type* (*WT*) mice were treated with IFN-β for 1.5h. Cells were cross-linked, sonicated, and immunoprecipitated with an IRF9-specific antibody and re-immunoprecipitated with a STAT1 or STAT2-specific antibody. The amount of precipitated DNA was measured by RT-qPCR. Data represent mean and SEM values of three independent experiments. P-values were calculated using the paired ratio t-test (*P ≤ 0.05; **P ≤ 0.01, ***P ≤ 0.001). **b, c)** Logos representing the motifs identified in the sequences underlying the STAT2-IRF9 peaks in untreated cells (b) and STAT1, STAT2-IRF9 and STAT1-STAT2-IRF9 (ISGF3) (c) peaks in IFN-β treated BMDM using de novo Meme searches.

Supplementary Fig. 4

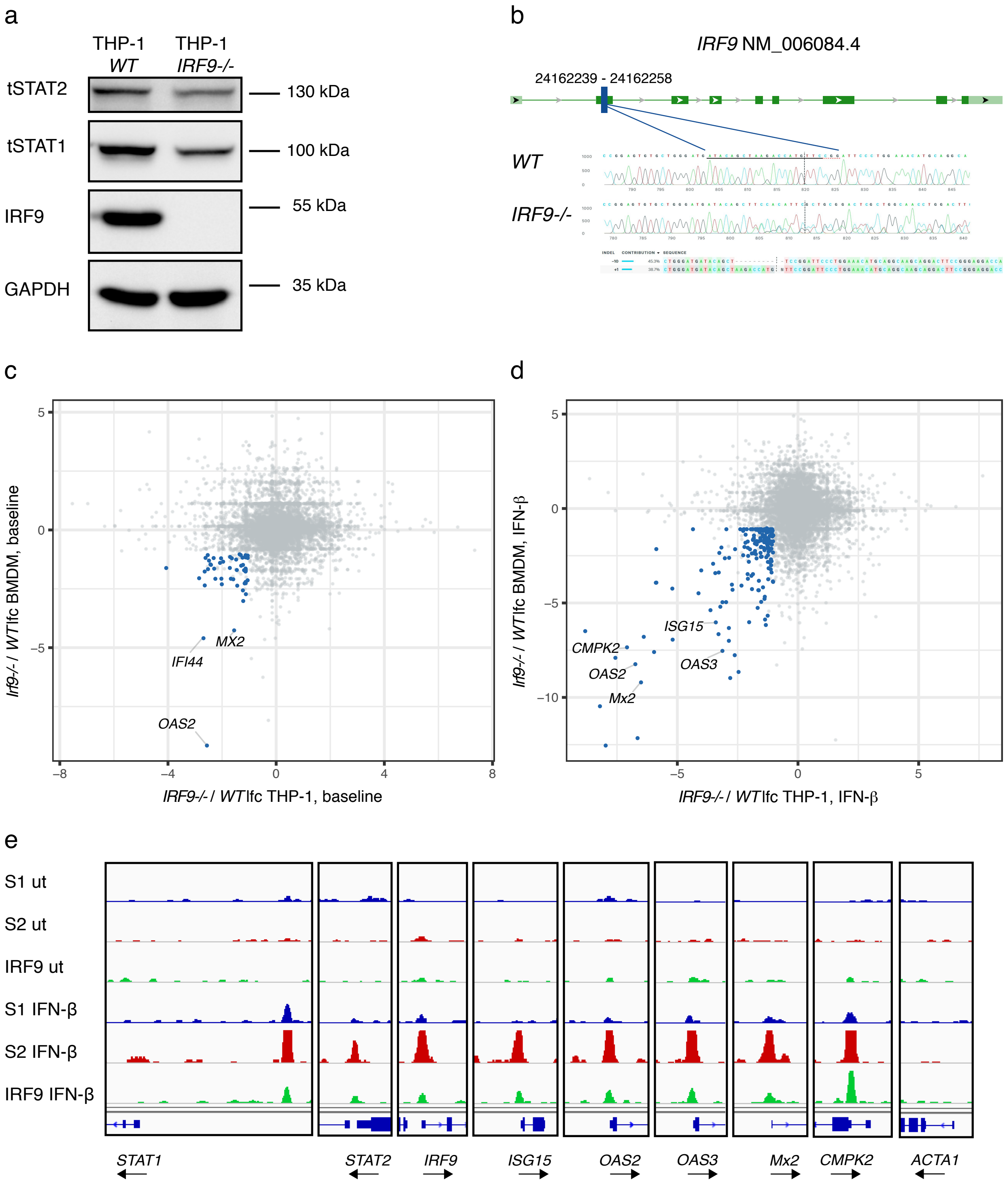

Supplementary Figure 4. IFN-induced changes in gene expression and chromatin-associated STAT complexes in human THP-1 cells, comparison to mouse BMDM. **a)** Western blot confirmation of IRF9 protein knockout in human monocytic THP-1 cells (single clone). **b)** Sequence analysis of an *IRF9-/-* THP-1 single clone. The composition and frequency of insertions and deletions (INDELS) was analyzed using ICE software (Synthego; https://ice.synthego.com/#/). **c, d)** Lfc/lfc plot comparing *WT* or *Irf9-/-* BMDM to human monocytic THP-1 cells. mRNA expression (n=3) ratios with a cutoff padj ≤ 0.05 und lfc ≥ 1 (*IRF9-/- / WT*) in resting (c) or in IFN-β treated (d) cells are plotted. Genes affected by the loss of IRF9 in both THP-1 and BMDM are displayed in blue. **e)** Representative genome browser tracks for transcription factor binding at ISG and control loci (scale 0-70) for STAT1 (S1/blue), STAT2 (S2/red) and IRF9 (green). Tracks represent ChIP-seq experiments in untreated and IFN-β treated THP-1 cells.Source data are provided as a Source Data file.

Supplementary Fig. 5

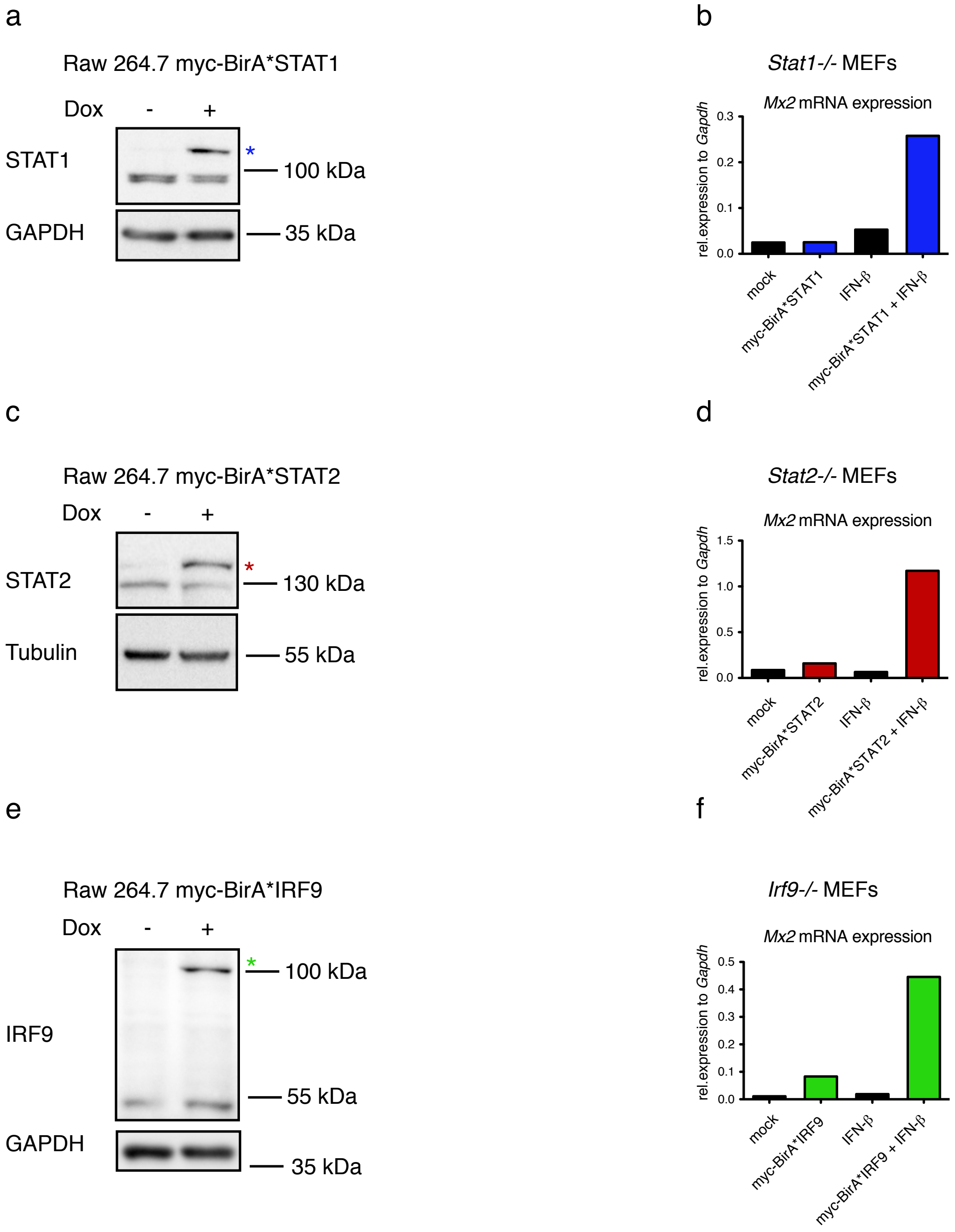

Supplementary Figure 5. a, c, e) Myc-BirA*-STAT1 (a), myc-BirA*-STAT2 (c) and myc-BirA*-IRF9 (e) transgenic Raw 264.7 cells were treated with 0,2μg/ml doxycycline. Whole-cell extracts were collected and tested in western blot for levels of STAT1, STAT2, IRF9, Tubulin and GAPDH. **b, d, f)** *Stat1-/-* (b), *Stat2-/-* (d), and *Irf9-/-* (f) mouse embryonic fibroblasts (MEFs) were transiently transfected with the indicated expression vectors. One day after transfection, cells were stimulated with IFN-β, RNA was isolated and *Mx2* expression determined by RT-qPCR. One representative of two independent experiments is shown.Source data are provided as a Source Data file.

Supplementary Fig. 6

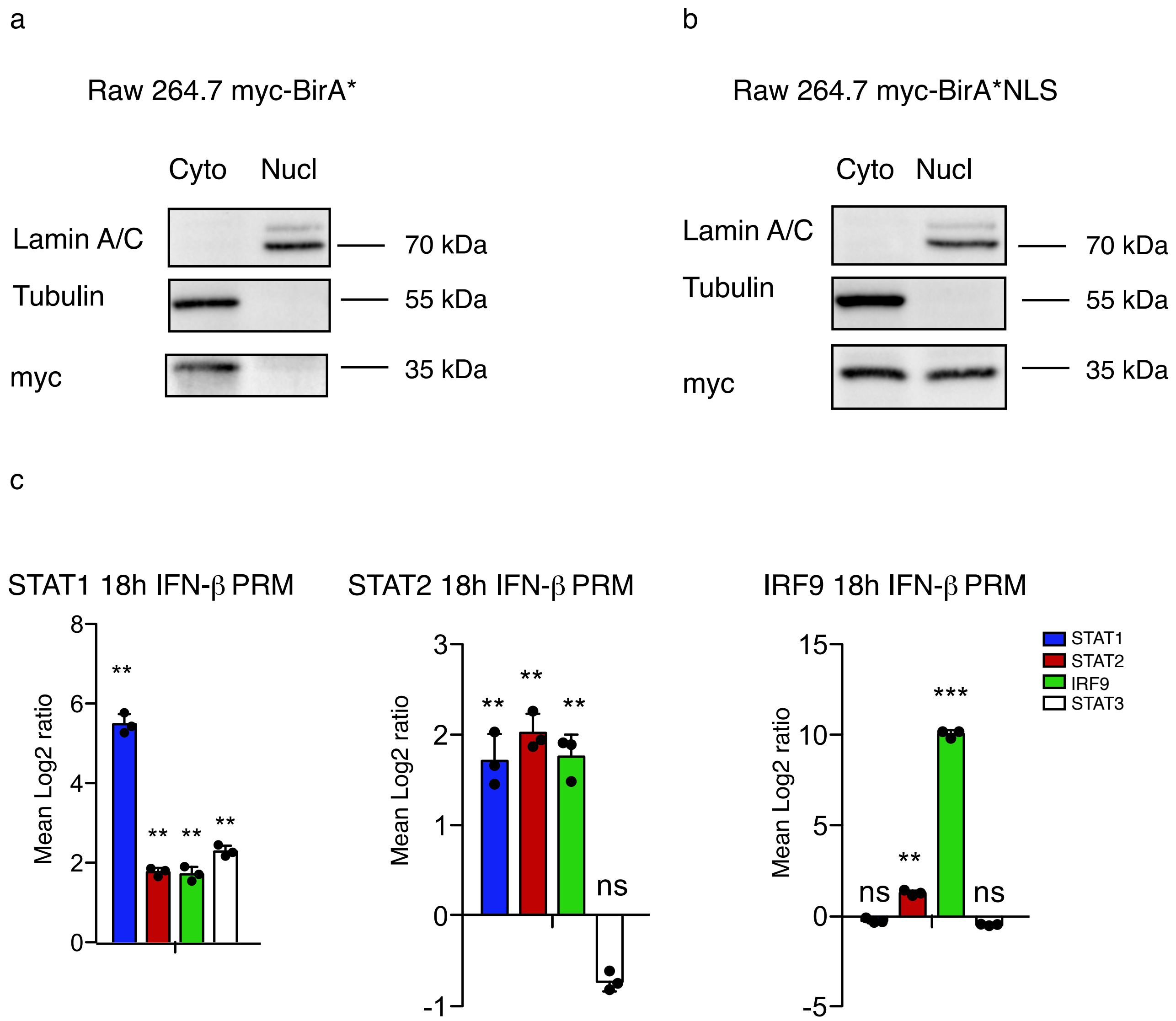

Supplementary Figure 6. a,b) Subcellular localization of myc-BirA* and myc-BirA*-NLS controls. Nuclear and cytoplasmic extracts from doxycycline induced myc-BirA* and myc-BirA*-NLS Raw 264.7 cells were analyzed by western blot. A 2:1 ratio of the nuclear to cytoplasmic fraction is shown. **c)** Targeted MS analysis of STAT1, STAT2 and IRF9 BioIDs using PRM. Raw 264.7 cells were treated with 0,2μg/ml doxycycline for 24h, followed by addition of 50μM biotin and IFN-β for 18 hours. Cells were lysed and protein complexes isolated by streptavidin affinity purification, followed by analysis with LC-MS. Mean Log2-transformed protein ratios were calculated for three biological replicates of myc-STAT1-BirA*, myc-STAT2-BirA* or myc-IRF9-BirA* cells normalized to their myc-BirA*-NLS control cells treated for 18h with IFN-β.

Standard deviation and t-test statistics were calculated for each of the target proteins. P-values (*P ≤ 0.05; **P ≤ 0.01, ***P ≤ 0.001).Source data are provided as a Source Data file.

Supplementary Fig. 7

a

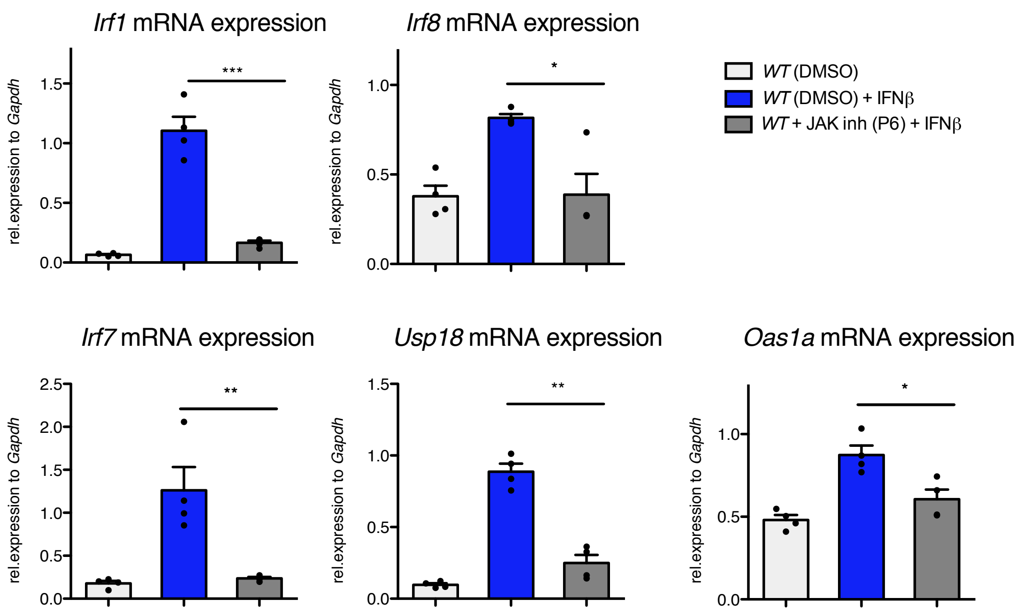

Supplementary Figure 7. a) BMDM isolated from *wild type* mice were left untreated (DMSO) or treated with 15μM P6 inhibitor for 3h followed by addition of IFN-β for 2 hours. *Gapdh*-normalized gene expression was measured by RT-qPCR. Data represent the mean and standard error of the mean (SEM) values of four independent experiments. P-values were calculated using the paired ratio t-test (*P ≤ 0.05; **P ≤ 0.01, ***P ≤ 0.001). Source data are provided as a Source Data file.

Supplementary reference

1. Cuartero, S. *et al.* Control of inducible gene expression links cohesin to hematopoietic progenitor self-renewal and differentiation. *Nat. Immunol.* **19,** 932–941 (2018).
