# Supplementary material for: A molecular switch from STAT2-IRF9 to ISGF3 underlies interferon-induced gene transcription
Source: Nat Commun. 2019 Jul 2;10:2921. doi: 10.1038/s41467-019-10970-y (PMC6606597; doi:10.1038/s41467-019-10970-y)
Supplement: Supplementary file 3 — Description of Additional Supplementary Files [file 41467_2019_10970_MOESM3_ESM.docx]

**Description of Supplementary Files**

**File Name:** **Supplementary Data 1**

**Description:** Genes bound by STAT1, STAT2-IRF9 and ISGF3 transcription factor complexes.

**File Name:** **Supplementary Data 2**

**Description:** Genes shown in the lfc/lfc plot of Fig. 3a comparing WT or Irf9-/- genotypes in BMDM and mouse embryonic fibroblasts (MEF). mRNA expression (n=3) ratios, with a cutoff padj ≤ 0.05 und lfc ≥ 1, (Irf9-/- / WT) in resting cells are shown.

**File Name:** **Supplementary Data 3**

**Description:** Genes shown in the lfc/lfc plot of Fig. 3b comparing IFN-β-treated cells with WT or Irf9-/- genotypes for BMDM and mouse embryonic fibroblasts (MEF). mRNA expression (n=3) ratios, with a cutoff padj ≤ 0.05 und lfc ≥ 1, (Irf9-/- / WT) are shown.

**File Name:** **Supplementary Data 4**

**Description:** Genes shown in the lfc/lfc plot of supplementary Fig. 4c comparing WT or Irf9-/- BMDM to wt or IRF9-/- human THP-1 cells. mRNA expression (n=3) ratios, with a cutoff padj ≤ 0.05 und lfc ≥ 1, (Irf9-/- / WT) in resting cells are shown.

**File Name:** **Supplementary Data 5**

**Description:** Genes shown in the lfc/lfc plot of supplementary Fig. 4d comparing IFN-β-treated WT or Irf9- /- mouse BMDM to IFN-β-treated wt or IRF9-/- human THP-1 cells. mRNA expression (n=3) ratios, with a cutoff padj ≤ 0.05 und lfc ≥ 1, (IRF9-/- / WT) are shown.

**File Name:** **Supplementary Data 6**

**Description:** PRM analysis, mass spectrometry.

**File Name:** **Supplementary Data 7**

**Description:** Shotgun proteomics data of STAT1, STAT2, and IRF9 BioID samples, MaxQuant search results and LIMMA statistics.

**File Name:** **Supplementary Data 8**

**Description:** Functional annotation of STAT1, STAT2 and IRF9 BioID interactors.
