# Supplementary material for: A molecular switch from STAT2-IRF9 to ISGF3 underlies interferon-induced gene transcription
Source: Nat Commun. 2019 Jul 2;10:2921. doi: 10.1038/s41467-019-10970-y (PMC6606597; doi:10.1038/s41467-019-10970-y)
Supplement: Supplementary file 12 — Reporting summary [file 41467_2019_10970_MOESM12_ESM.pdf]

nature research

Corresponding author(s): Thomas Decker

Last updated by author(s): Mar 25, 2019

Reporting Summary

Nature Research wishes to improve the reproducibility of the work that we publish. This form provides structure for consistency and transparency in reporting. For further information on Nature Research policies, see Authors & Referees and the Editorial Policy Checklist.

Statistics

For all statistical analyses, confirm that the following items are present in the figure legend, table legend, main text, or Methods section.

n/a	Confirmed	
☐	☒	The exact sample size (*n*) for each experimental group/condition, given as a discrete number and unit of measurement
☐	☒	A statement on whether measurements were taken from distinct samples or whether the same sample was measured repeatedly
☐	☒	The statistical test(s) used AND whether they are one- or two-sided<br>*Only common tests should be described solely by name; describe more complex techniques in the Methods section.*
☒	☐	A description of all covariates tested
☐	☒	A description of any assumptions or corrections, such as tests of normality and adjustment for multiple comparisons
☐	☒	A full description of the statistical parameters including central tendency (e.g. means) or other basic estimates (e.g. regression coefficient) AND variation (e.g. standard deviation) or associated estimates of uncertainty (e.g. confidence intervals)
☐	☒	For null hypothesis testing, the test statistic (e.g. *F*, *t*, *r*) with confidence intervals, effect sizes, degrees of freedom and *P* value noted<br>*Give P values as exact values whenever suitable.*
☒	☐	For Bayesian analysis, information on the choice of priors and Markov chain Monte Carlo settings
☐	☒	For hierarchical and complex designs, identification of the appropriate level for tests and full reporting of outcomes
☒	☐	Estimates of effect sizes (e.g. Cohen's *d*, Pearson's *r*), indicating how they were calculated

Our web collection on statistics for biologists contains articles on many of the points above.

Software and code

Policy information about availability of computer code

Data collection	Western blot images were developed using Bio-Rad Chemidoc touch and further analysed with Image Lab version 5.2.1, Bio-Rad,<br>ChIP and RNA-seq samples were run on HiSeqV4 SR50<br>Cycle threshold (Ct) values of mRNA qPCR data were measured using Eppendorf Realplex 2 Mastercycler.
Data analysis	Perseus 1.5.5.3, (Tyanova et al., 2016), http://www.coxdocs.org/doku.php?id=perseus:start<br>Skyline, (MacLean et al., 2010), https://skyline.ms/project/home/begin.view?<br>LIMMA package, http://bioconductor.org/packages/release/bioc/html/limma.html<br>MaxQuant software package, https://www.biochem.mpg.de/5111795/maxquant<br>ImageJ32 for Mac, https://imagej.nih.gov/ij/<br>Image Lab version 5.2.1, Bio-Rad<br>GraphPad Prism, www.graphpad.com<br>bwa/0.7.12 alignment, http://bio-bwa.sourceforge.net/<br>samtools/1.3.1, http://samtools.sourceforge.net/<br>TopHat v2.1.1, https://ccb.jhu.edu/software/tophat/index.shtml<br>htseq-count v0.6.1. https://htseq.readthedocs.io/en/release_0.10.0/<br>AQUAS TF pipeline, Kundaje Lab, https://github.com/kundajelab/chipseq_pipeline<br>BWA (v0.7.13), http://bio-bwa.sourceforge.net/<br>Picard MarkDuplicates (v1.126), Broad Institute, https://broadinstitute.github.io/picard/<br>macs2 (v2.1.1), https://github.com/taoliu/MACS<br>spp (v1.13), https://www.encodeproject.org/software/spp/<br>bedtools; v.25.0, https://bedtools.readthedocs.io/en/latest/content/tools/intersect.html<br>picard; v2.1.1; MarkDuplicates, https://software.broadinstitute.org/gatk/documentation/tooldocs/current/<br>picard_sam_markduplicates_MarkDuplicates.php<br>deeptools; v3.1.1, https://deeptools.readthedocs.io/en/develop/<br>DESeq 2 v1.16.11, https://bioconductor.org/packages/release/bioc/html/DESeq2.html

TopHat v2.1.1, https://ccb.jhu.edu/software/tophat/index.shtml

For manuscripts utilizing custom algorithms or software that are central to the research but not yet described in published literature, software must be made available to editors/reviewers. We strongly encourage code deposition in a community repository (e.g. GitHub). See the Nature Research guidelines for submitting code & software for further information.

Data

Policy information about availability of data

All manuscripts must include a data availability statement. This statement should provide the following information, where applicable:
- Accession codes, unique identifiers, or web links for publicly available datasets
- A list of figures that have associated raw data
- A description of any restrictions on data availability

Raw and analyzed data reported in this paper are available under accession number GEO: GSE115435 [https://www.ncbi.nlm.nih.gov/geo/query/acc.cgi?acc=GSE115435]. The mass spectrometry proteomics data have been deposited to the ProteomeXchange Consortium (http://proteomecentral.proteomexchange.org) via the PRIDE partner repository 80 with the dataset identifier PXD013209 [http://proteomecentral.proteomexchange.org/cgi/GetDataset?ID=PXD013209] for the shotgun (interactome) dataset or via Panorama Public 81 with the identifier PXD013251 [http://proteomecentral.proteomexchange.org/cgi/GetDataset?ID=PXD013251
] in case of the targeted MS data.

Field-specific reporting

Please select the one below that is the best fit for your research. If you are not sure, read the appropriate sections before making your selection.

☒ Life sciences ☐ Behavioural & social sciences ☐ Ecological, evolutionary & environmental sciences

For a reference copy of the document with all sections, see nature.com/documents/nr-reporting-summary-flat.pdf

Life sciences study design

All studies must disclose on these points even when the disclosure is negative.

Sample size	No sample- size calculation was performed.
Data exclusions	No data was excluded from experiments and analysis
Replication	Most data were repeated at least three times and the the data were reproducible.
Randomization	Cells were plated and distributed at equal density for treatment and control groups. The confluence of the cells at the time of treatment was noted to be equal and the allocation of treatment was randomly assigned.
Blinding	N.a.

Reporting for specific materials, systems and methods

We require information from authors about some types of materials, experimental systems and methods used in many studies. Here, indicate whether each material, system or method listed is relevant to your study. If you are not sure if a list item applies to your research, read the appropriate section before selecting a response.

Materials & experimental systems

n/a	Involved in the study	
☐	☒	Antibodies
☐	☒	Eukaryotic cell lines
☒	☐	Palaeontology
☐	☒	Animals and other organisms
☒	☐	Human research participants
☒	☐	Clinical data

Methods

n/a	Involved in the study	
☐	☒	ChIP-seq
☒	☐	Flow cytometry
☒	☐	MRI-based neuroimaging

Antibodies

Antibodies used	Stat1 (E-23) (used in ChIP for BMDM and MEF) Santa Cruz sc-346, RRID:AB_632435<br>Stat2 (L-20) (used in ChIP for BMDM and MEF) Santa Cruz sc-950, RRID:AB_2271322<br>Stat1 (western blot) Cell Signaling Catalog #9172, RRID:AB_2198300<br>Stat2 (D9J7L) (western blot) Cell Signaling Catalog #72604

α-Tubulin (DM1A) (western blot) Sigma Catalog #T9026
Phospho-Stat1 (Tyr701) (western blot) Cell Signaling Catalog #9167, RRID:AB_561284
Phospho-STAT2 (Tyr689) (western blot) Merck Catalog #07-224, RRID:AB_2198439
Lamin A/C (E-1) (western blot) Santa Cruz Catalog #sc-376248, RRID:AB_10991536
GAPDH (western blot) Millipore Catalog #ABS16,RRID:AB_10806772
IRF9 (6F1) (western blot, ChIP for BMDM and MEF, IF, IP)
Alexa Fluor 488 IgG (H+L) Thermo Scientific Catalog # A-11001, RRID:AB_2534069
Peroxidase-conjugated AffiniPure Goat Anti-Rabbit IgG (H+L) Jackson ImmunoResearch Inc. Code # 111-035-003
Peroxidase-conjugated AffiniPure Goat Anti-Mouse IgG (H+L) Jackson ImmunoResearch Inc. Code # 115-035-003
STAT1(ChIP-seq in THP-1 and IP) Cell Signaling Catalog #14995
STAT2 (ChIP-seq in THP-1 and IP)Cell Signaling Catalog # 72604
IRF9 (ChIP-seq in THP1)Cell Signaling Catalog # 76685
IgG (IP) Cell Signaling, Catalog #3900S

Validation	The antibodies were validated by western blot (correct molecular weight, expected response to physiological stimuli) and ChIP-qPCR in corresponing knock-out cells.

Eukaryotic cell lines

Policy information about cell lines

Cell line source(s)	Raw 264.7 cells (ATCC #TIB-71), THP-1 cells (ATCC #TIB-202), MEFs were immortalized in our lab using the 3T3 protocol.
Authentication	RNA expression by RT-qPCR was used to confirm a macrophage-type response to infection and cytokine treatment. No other cell line authentications were performed.
Mycoplasma contamination	All cell lines tested negative for mycoplasma contamination.
Commonly misidentified lines (See ICLAC register)	N.a.

Animals and other organisms

Policy information about studies involving animals; ARRIVE guidelines recommended for reporting animal research

Laboratory animals	C57BL/6N, Irf9-/-, Stat1-/-, and Stat2-/- mice were backcrossed for more than 10 generations on a C57BL/6N background were housed in the same specific-pathogen-free (SPF) facility under identical conditions according to recommendations of the Federation of European Laboratory Animal Science Association and additionally monitored for being norovirus negative.
Wild animals	The study did not involve wild animals.
Field-collected samples	N.a.
Ethics oversight	Animal experiments were approved by the institutional ethics and animal welfare committee of the University of Veterinary Medicine, Vienna, and the national authority (Austrian Federal Ministry of Education, Science and Research) according to §§26ff of Animal Experiments Act (Tierversuchsgesetz TVG 2012, BGBl. I Nr 114/2012) under the permission license numbers BMWF 68.205/0032-WF/II/3b/2014 and BMWFW-68.205/0212-WF/V/3b/2016. Animal husbandry and experimentation was performed under the Austrian national law and the ethics committees of the University of Veterinary Medicine Vienna and according to the guidelines of FELASA which match those of ARRIVE.

Note that full information on the approval of the study protocol must also be provided in the manuscript.

ChIP-seq

Data deposition

☒ Confirm that both raw and final processed data have been deposited in a public database such as GEO.

☒ Confirm that you have deposited or provided access to graph files (e.g. BED files) for the called peaks.

Data access links<br>*May remain private before publication.*	Raw and analyzed data reported in this paper are available under accession number GEO: GSE115435 [https://www.ncbi.nlm.nih.gov/geo/query/acc.cgi?acc=GSE115435]
Files in database submission	GSE115433 STAT1, STAT2 and IRF9 transcription factor binding analysis in wild type and Irf9-/- bone marrow derived macrophages in response to type I and type II interferons<br><br>GSE128107 STAT1, STAT2 and IRF9 transcription factor binding analysis in wild type mouse embryonic fibroblasts (MEF) in response to type I interferons<br><br>GSE128111 STAT1, STAT2 and IRF9 transcription factor binding analysis in wild type human monocytic THP1 cells in response to type I interferons

Genome browser session
(e.g. UCSC)

N. a.

Methodology

Replicates	For ChIP-seq in BMDM we used two biological replicates, for THP-1 and MEF one biological replicate from pooled cells.
Sequencing depth	We used 50-bp, single-end reads and sequencing depth was between 10-40 million reads.
Antibodies	Stat1 (E-23) (used in ChIP for BMDM and MEF) Santa Cruz sc-346, RRID:AB_632435<br>Stat2 (L-20) (used in ChIP for BMDM and MEF) Santa Cruz sc-950, RRID:AB_2271322<br>IRF9 (6F1) (western blot, ChIP for BMDM and MEF, IF, IP)<br>STAT1(ChIP-seq in THP-1 and IP) Cell Signaling Catalog #14995<br>STAT2 (ChIP-seq in THP-1 and IP)Cell Signaling Catalog # 72604<br>IRF9 (ChIP-seq in THP1)Cell Signaling Catalog # 76685
Peak calling parameters	Peak Calling using macs2 (v2.1.1) and spp (v1.13).
Data quality	Data quality of all sequencing runs were confirmed by FastQC.
Software	Same as "Software and code" section here above
